# Locus of control and subjective well-being: Panel evidence from Australia

**DOI:** 10.1371/journal.pone.0272714

**Published:** 2022-08-31

**Authors:** Dusanee Kesavayuth, Dai Binh Tran, Vasileios Zikos

**Affiliations:** 1 Faculty of Economics, Department of Economics, Kasetsart University, Bangkok, Thailand; 2 Faculty of Economics and Management, Vietnamese-German University, Thu Dau Mot City, Binh Duong Province, Vietnam; 3 Faculty of Economics, Chulalongkorn University, Pathumwan, Bangkok, Thailand; Groupe ESC Dijon Bourgogne, FRANCE

## Abstract

This paper’s aim is to propose a mediation framework and test whether lifestyle choices and social capital are pathways through which locus of control (LoC) affects subjective well-being. Using longitudinal data for Australia, we find that life satisfaction and mental health are explained by direct and indirect effects of LoC. The direct effect is positive, indicating that individuals with an internal LoC have higher levels of life satisfaction and mental health. We also show that physical activity and social interaction are two pathways linking an internal LoC to higher levels of well-being. Our findings provide new insights into the relationship between LoC and subjective well-being and suggest that, if the aim of policy is to improve well-being, the focus should be on enabling people to develop an internal LoC. This may lead to higher well-being both through the identified channels and, more importantly, through the direct channel of LoC.

## 1. Introduction

Non-cognitive skills, such as the Big Five personality traits (agreeableness, conscientiousness, extroversion, emotional stability and openness), predict a variety of labor market and behavioral outcomes (e.g., [1–4]). Non-cognitive skills are also intricately intertwined with well-being, influencing how people’s well-being responds to various life events [5–9].

This paper focuses on one specific non-cognitive skill–locus of control (LoC). LoC is often conceptualized as a form of human capital accumulated through parental involvement and investments in education [10]. The remaining proportion of the variation in LoC, which ranges between 30 and 50%, is attributed to genetic factors [11,12]. LoC reflects “whether or not the person perceives a causal relationship between his own behavior and the reward” [13]. Individuals who believe that they are in control of life’s outcomes and their own destinies have an *internal* LoC. In contrast, those who believe that what happens in life stems mainly from external factors, such as fate, luck or other people, have an *external* LoC [10,14].

More and more empirical evidence is highlighting the importance of LoC for behavioral and economic outcomes. For example, [15] and [16] show that individuals with an internal LoC are more likely to graduate from high school. Individuals with an internal LoC are also more likely to eat healthily, exercise more and smoke less [17,18]; have better health outcomes [19,20]; are more likely to acquire productive skills [1]; are more willing to assume financial risk [21]; and devote more effort to job search [22].

Economists and other social scientists increasingly recognize the role of LoC for maintaining high levels of well-being. In a recent study, [23] investigate whether the gender gap in mental health could be explained by gender differences in LoC. They use longitudinal data from the Household, Income and Labour Dynamics in Australia (HILDA) survey as we do here. With the aid of the Blinder-Oaxaca decomposition and alternative approaches, their findings suggest that gender differences in LoC contribute to explaining the gender gap in mental health. If women and men were the same in terms of their LoC, the gender gap in mental health could be reduced by 18.8%. This is an important result and suggests that education programs that teach positive control beliefs to children should be designed particularly with girls in mind.

The finding that having an internal LoC is associated with higher levels of well-being has also been documented in other studies. For example, individuals with an internal LoC are more likely to be psychologically insured against negative life events [9], and have a lower risk of experiencing psychological distress and depression [19,24]. Having an internal LoC has also been shown to promote the well-being of managers [25]. [26] conducted an in-depth meta-analysis and estimated a correlation of about 0.32 between internal perceptions of control and job satisfaction.

What explains the relationship between LoC and well-being? While a large body of literature has examined the direct effects of LoC, little is known about the indirect, mediational pathways linking LoC to well-being. In this study we consider the role of two forms of human capital investments as possible mediators: lifestyle choices and social capital. Both forms of human capital are worth investigating because they are important determinants of a person’s well-being and relevant to welfare policies.

There is a considerable literature showing that LoC plays an important role for the health investments that individuals make. For example, relative to those with an external LoC, those with an internal LoC tend to be more physically active [17,18]. At the same time, physical activity is an important factor contributing to a person’s well-being. Evidence suggests that physical activity and exercise have various anxiolytic and antidepressive benefits that may help increase well-being [27,28]. In light of these findings, we utilize information on physical activity and time spent on outdoor tasks as indicators of lifestyle choices that may be linked with both LoC and well-being.

Besides lifestyle choices, evidence suggests that LoC matters for the extent to which individuals invest in social capital. Studies in this area have shown that those with an internal LoC tend to be more sociable [9,18]. Moreover, there is a strong link between social capital and well-being. Studies have found that spending time with family and friends promotes a sense of well-being (e.g., [29,30]). Conversely, individuals with low levels of social capital may often experience feelings of social isolation and loneliness. Those individuals are more likely to develop psychological distress and depression [31]. Based on these findings, we utilize information on social interactions, participation in volunteer work, and whether the individual is an active member of a social club as indicators of a person’s social capital.

Overall, the existing empirical literature suggests that (i) LoC is an important factor contributing to higher levels of well-being; (ii) LoC is positively associated with healthy behaviors and social capital; and (iii) healthy behaviors and social capital promote well-being. However, the earlier studies have not drawn the link between these three findings. Ours is the first study to include all three possible relationships in the same model, potentially providing new insights both on *whether* LoC matters for well-being and *why* it matters. Figure A1 in the S1 Appendix provides a simple graphical representation of how LoC relates to well-being.

Building upon the earlier findings, we hypothesize that individuals who feel more in control of life’s outcomes enjoy higher levels of well-being through the direct channel of LoC. They may also enjoy higher well-being through indirect channels. For the direct effect, we model well-being as a function of LoC, controlling for confounders. For the indirect effects, we consider the role of certain lifestyle choices and social capital. Accordingly, we quantify both the direct effect of LoC on well-being and the indirect, mediational pathways [32–34] discuss methodological contributions on mediation analysis. For economic studies in this area see, for example, [35–41]. As well as being significant from an empirical point of view, our findings may be useful particularly for policy makers who may wish to enhance well-being.

To preface our results, we find that LoC has a significant direct and indirect impact on life satisfaction and mental health. The direct effect is positive, indicating that individuals with an internal LoC have higher levels of life satisfaction and mental health. We also show that physical activity and social interaction are two pathways through which LoC leads to higher well-being. Our findings suggest that policies and intervention programs aimed at improving well-being should focus on enabling people to develop an internal LoC. Such initiatives may lead to higher well-being both through the identified channels and, more importantly, through the direct channel of LoC or via other unobserved pathways.

## 2. Data

Our data are drawn the Household Income and Labour Dynamics in Australia (HILDA) survey. HILDA collects longitudinal information from a large, nationally representative sample of Australian households. The survey started in 2001 with participation of almost 14,000 individuals from 7,682 households. All household members aged 15 or older are interviewed through both face-to-face interviews and self-completion questionnaires. [42] provide detailed information on the HILDA survey.

Our analytical sample consists of individuals 15–75 years old. Data on LoC are available in the 2003, 2004, 2007, 2011 and 2015 waves of the HILDA survey. We use data for LoC from those waves and data for the other variables included in our analysis from 2004, 2005, 2008, 2012 and 2016. After excluding observations with missing answers to the questions required for our analysis, the final sample corresponded to an unbalanced panel of 17,428 individuals and 41,696 observations. We note that this study has been exempted from ethics review (No. 038/63) by The Research Ethics Review Committee for Research Involving Human Research Participants, Group I, Chulalongkorn University. All procedures performed in studies involving human participants were in accordance with the ethical standards of the institutional and/or national research committee and with the 1964 Helsinki declaration and its later amendments or comparable ethical standards.

### 2.1 Well-being measures

Well-being is captured using two separate measures. The first is a single-item measure of life satisfaction, while the second measure is based on a series of questions about mental health. The life satisfaction question asks: “All things considered, how satisfied are you with your life?” Answers are reported on an 11-point scale that ranges between 0 (totally dissatisfied) and 10 (totally satisfied). The life satisfaction measure is often used in the literature and has been shown to have a number of desirable features including validity, high test-retest reliability and stability [43,44].

To measure mental health, we employ the five-item Mental Health Inventory (MHI-5) scale, which is a subscale of the 36-item Short Form Health Survey (SF-36). The MHI-5 scale is frequently used by economists and other social scientists and is considered a good proxy for a person’s mental well-being (e.g., [23,45,46]). Individuals were asked “How much of the time during the past four weeks: (a) Have you been a very nervous person; (b) Have you felt so down in the dumps that nothing could cheer you up; (c) Have you felt calm and peaceful; (d) Have you felt downhearted and blue; (e) Have you been a happy person?” Possible responses range from 1 (none of the time) to 6 (all the time). The HILDA survey provides the mental health score on a 0–100 scale, where higher values represent higher levels of mental health.

### 2.2 The LoC measure

HILDA uses the Psychological Coping Resources component of the Mastery Module developed by [47]. This is intended to capture self-efficacy rather than LoC as developed by [13]. Given that the two scales exhibit substantial overlap and are often assumed to be interchangeable [17], in our analysis we use the term “LoC” for clarity.

Information on LoC is drawn from responses to seven items. The items are: (1) I have little control over the things that happen to me; (2) There is really no way I can solve some of the problems I have; (3) There is little I can do to change many of the important things in my life; (4) I often feel helpless in dealing with the problems of life; (5) Sometimes I feel that I’m being pushed around in life; (6) What happens to me in the future mostly depends on me; and (7) I can do just about anything I really set my mind to do. Possible answers range from 1 (strongly disagree) to 7 (strongly agree).

To explore the underlying structure of the seven LoC items, we use factor analysis confirming a two-factor solution: items (6) and (7) load onto one factor (internal LoC), while the remaining items load onto another factor (external LoC). We note that a test of internal consistency yields a Cronbach’s alpha reliability statistic of 0.84, thus allowing us to construct a single LoC index, similar to past studies in the literature [9,18,21,22,48]. Accordingly, we reverse the scores of items 1–5, and then add the scores of all items, 1 through 7. The total score thus ranges from 7 to 49 with higher values representing a higher (more internal) LoC.

### 2.3 Other explanatory variables

Our analysis includes a range of standard socio-economic controls to account for the potentially confounding effects of variables that may be correlated simultaneously with a person’s LoC and his/her well-being. These include age and its squared term, household size, real household income, educational attainment, employment status, marital status, negative life events, Australian states of residence and territories, and time (waves). Table A1 in the S1 Appendix provides summary statistics of all the variables included in our analysis.

## 3. Empirical model

Let *W*_*it*_ be a particular well-being measure, life satisfaction or mental health, of individual *i* at time *t*. Our empirical model is specified as follows:

$$W_{it}=a_{0}+{a_{1}LoC}_{i,t-1}+a_{2}X_{it}+a_{3}T_{t}+\mu_{i}+\varepsilon_{it}$$ (1)

where *LoC*_*i*,*t*−1_ is the one-period lagged value of LoC; *X*_*it*_ is a vector of time-varying predictor variables including regional dummies; *T*_*t*_ is a vector of wave dummies; *μ*_*i*_ is the person-specific error; and *ε*_*it*_ is the idiosyncratic error.

One issue with Eq (1) is that LoC is likely endogenous. Using the HILDA dataset, [10] conducted an in-depth analysis of the stability of LoC. They found that LoC does not vary substantially in the Australian adult population and is generally unaffected by changes in demographic, labor market, and health events.

To investigate the stability of LoC in our data, we report results from the transition matrix, which displays the mean transition probability for all waves *t*-1 to *t* across all individuals. Looking across the columns of Table 1, we can see that if an individual has LoC score 7–13 in period *t*-1, the probability that he/she remains within the same range of LoC values in period *t* is 27.38%. For this individual, the probability of having LoC 21–27 in period *t* is 22.62%, while there is a 9.52% probability of having LoC 28–34.

**Table 1:** Transition matrix for LoC.

	LC range (t)					
LC range (t-1)	7–13	14–20	21–27	28–34	35–41	41–49
7–13	27.38	27.38	22.62	9.52	8.33	4.76
14–20	5.81	20.1	32.93	23.49	8.96	8.72
21–27	1.09	7.37	27.2	35.83	20.37	8.14
28–34	0.3	2.22	13.88	34.7	32.52	16.38
35–41	0.15	0.7	4.51	19.69	41.85	33.11
41–49	0.11	0.43	1.77	7.68	24.65	65.38

Note: The total number of observations is 41,696 across 17,428 individuals.

There is even more variation for LoC scores 14–20 or higher. For example, an individual who scores 14–20 on LoC in period *t*-1 has a 20.1% probability to be within the same range of LoC values in period *t*. Interestingly, the same individual has a much higher probability at 32.93% of transitioning to the immediately higher range of LoC values, 21–27 (but only a 5.81% probability of moving to the immediately lower range, 7–13). Likewise, for an individual with LoC score 21–27, the probability that he/she will transition to the immediately higher category of 28–34 is 35.83%. Similar results are obtained for those with a more internal sense of control, thus providing additional evidence that LoC may change over time.

Estimation of Eq (1) requires dealing with person-specific unobserved heterogeneity. For longitudinal data, this can be done using a fixed effects model. While it is true that only random effects can be used to estimate the coefficients of variables that are time-invariant at the individual level, this is done at the cost of assuming zero correlations between the explanatory variables *X*_*it*_ and the person-specific error *μ*_*i*_. The fixed effects model, on the other hand, avoids this source of potential bias in the estimates by allowing for correlations between *X*_*it*_ and *μ*_*i*_, thus controlling for the effects of time-invariant unobservables.

Another advantage of using the fixed effects model, and thus relying on within-person variation to estimate the coefficient on LoC, is that different people may interpret subjective well-being questions differently. We may not be able to say that an individual who reports life satisfaction of 8 is happier than someone who reports a 7, but it is more reasonable to assume that a person who reports 7 in one year and 8 in the next year has experienced an increase in their life satisfaction. A fixed effects model can help to avoid such bias.

An additional empirical issue we need to deal with is the potential for reverse causality. It is quite plausible that some people will report a more internal LoC if they are having a good day, and hence one would observe a positive correlation between LoC and well-being just for this reason alone. To mitigate this concern, instead of using the contemporaneous value of LoC in Eq (1), we use the value lagged to period *t*-1. Although this approach does not permit estimation of the contemporaneous effect of LoC, it is reasonable to assume that the effects of LoC may take some time to feed through to higher well-being.

For the reasons indicated above, all our estimations are carried out using linear fixed-effects models with robust standard errors clustered at the individual level [49], although qualitatively similar conclusions can also be reached using an ordered logit model. To aid the interpretation of our results, we standardized both our well-being variables and the LoC variable to have mean 0 and standard deviation 1.

## 4. Results

Table 2 presents initial evidence of how LoC relates to life satisfaction and mental health. The estimates in column 1 suggest that LoC enters positively the life satisfaction regression equation. This implies that individuals with an internal LoC report higher levels of life satisfaction, consistent with previous findings in the literature [19,24]. A standard deviation increase in internal perceptions of control is associated with an approximately 0.111 standard deviation increase in life satisfaction. The coefficient estimate is statistically significant at p-values < 0.01.

**Table 2:** The effect of LoC on well-being.

	Life Satisfaction	Mental health
LoC _t-1_	0.111***	0.118***
	(0.0081)	(0.0078)
Age	0.0157	0.144***
	(0.4810)	(0.0209)
Age squared	0.0415***	0.0193***
	(0.0044)	(0.0043)
Household size	-0.0157***	-0.0182***
	(0.0060)	(0.0058)
Real household income	0.0438***	0.0236**
	(0.0107)	(0.0101)
College and above	-0.0376	-0.023
	(0.0275)	(0.0271)
Unemployed	-0.115***	-0.101***
	(0.0380)	(0.0341)
Not in the labor force	-0.013	-0.0625***
	(0.0182)	(0.0172)
Living as a couple	-0.0208	0.0104
	(0.0257)	(0.0240)
Separated	-0.285***	-0.151***
	(0.0505)	(0.0417)
Divorced	-0.160***	-0.0171
	(0.0501)	(0.0416)
Widowed	-0.317***	-0.157**
	(0.0818)	(0.0778)
Never married and not living as a couple	-0.187***	-0.0564*
	(0.0315)	(0.0312)
Individuals	17,428	17,428
Observations	41,696	41,696

Note

*p<0.1

**p<0.05

***p<0.01. Control variables include negative life events, Australian states of residence and territories, and waves. Robust standard errors are in parentheses.

Up until now the analysis has focused on life satisfaction as the measure of well-being. An advantage of the HILDA dataset is that it also contains a multi-item measure of mental health. Column 2 of Table 2 shows that, as for life satisfaction, LoC is significantly related to mental health. A standard deviation increase in internal sense of control is associated with a 0.118 standard deviation increase in mental health, which is statistically significant at the 1% level.

To better understand the magnitude of these estimates, take for example the average person in perceptions of control and the person who is one standard deviation above the average. The difference between these two groups of individuals, as Table 2 shows, is 0.111 standard deviations in life satisfaction. This effect is quite large. All things being equal, it is approximately the same as the effect of being unemployed versus being employed. The estimated effect is even more substantial when it comes to mental health. Here, we find that the difference between the average LoC person and the person who is one standard deviation above the average is 0.118. Such difference is about 1.2 times the effect that being unemployed has on mental health, ceteris paribus. The strong effects on life satisfaction and mental health highlight the importance of individuals’ LoC for their well-being.

## 5. Pathways

Our findings indicate that having an internal LoC is associated with higher levels of life satisfaction and mental health. In this section we investigate what pathways might help explain these relationships. Drawing on evidence from the existing literature, we analyze the role of two forms of human capital investments: lifestyle choices and social capital. Both forms of human capital are worth investigating because they are important determinants of a person’s well-being and relevant to welfare policies.

### 5.1 Do those with an internal LoC pursue a more active lifestyle?

Previous research suggests a positive relationship between LoC and health investments. For example, [17] showed that those with an internal LoC are more likely to engage in regular physical exercise. Other studies have highlighted physical activity as an important driver of well-being [27] concluded that exercise has various anxiolytic and antidepressive benefits, and research by [28] revealed that people who engage in physical activity are more likely to experience increased well-being.

Taken together, these results open up the possibility that individuals who feel more in control of life’s outcomes enjoy higher well-being simply because they pursue a more active lifestyle compared to their external counterparts.

To test whether LoC matters for the decision to be more active, we utilize standardized responses to a question about (i) the frequency of physical activity and (ii) the number of hours allocated to outdoor tasks in a typical week. The frequency of physical activity is taken from responses to the question: “In general, how often do you participate in moderate or intensive physical activity for at least 30 minutes?” Answers are on a 6-point scale that ranges from 0 (not at all) to 5 (every day). Individuals were also asked to indicate how much time they spend on outdoor tasks in a typical week.

Looking across the columns of Table 3, we can see that LoC is significantly associated with physical activity, indicating that those with an internal LoC tend to engage in physical activity more frequently. Interestingly, there is no relationship between LoC and the amount of time allocated to outdoor tasks. Overall, these results suggest that individuals with an internal LoC may enjoy higher levels of well-being because they participate more often in physical activity than individuals with an external LoC.

**Table 3:** The effect of LoC on lifestyle choices.

	Physical activity	Outdoor tasks
LoC _t-1_	0.0322***	0.0116
	(0.0076)	(0.0072)
Individuals	17,428	17,428
Observations	41,696	41,696

Note

*** p<0.01. Control variables include age, age squared, household size, real household income, educational attainment, employment status, marital status, negative life events, Australian states of residence and territories, and waves. Robust standard errors are in parentheses.

### 5.2 Do those with an internal LoC invest more in social capital?

It is now well established that social capital plays a vital role for subjective well-being. Several studies have found that time spent with friends and family is strongly linked to a person’s well-being (e.g., [29,30]). Another body of literature has highlighted that LoC matters for the extent to which individuals invest in social capital. Studies in this area have shown that individuals with an internal LoC tend to engage in more frequent socialization than those with an external locus of control [9,18].

Taken together, these findings raise the possibility that individuals with an internal sense of control invest more in social relationships, and this in turn may enhance their well-being. In other words, they may have higher well-being because they invest more in social capital.

To test this hypothesis, we analyze whether individuals’ LoC relates to their investments in social capital. Social capital is captured by three separate measures. The first is based on individuals’ responses to the question: “In general, about how often do you get together socially with friends or relatives not living with you?”, with possible answers ranging from 0 (less often than once every 3 months) to 6 (every day). The second measure is a binary indicator that takes the value 1 if the respondent is currently an active member of a sporting, hobby or community-based club or association, and 0 otherwise. The third measure is derived from responses to a question that asks individuals to indicate how much time they allocate in a typical week for volunteer/charity work. This measure, along with our first measure on the frequency of getting together socially with friends or relatives, are standardized to mean 0 and standard deviation 1.

The estimates in Table 4 suggest that those with an internal LoC tend to see their friends or relatives more often, consistent with [9] and [18]. They are also more likely to be active members of a social club. However, there is no evidence that LoC is associated with the amount of time allocated to volunteer/charity work. All in all, these results suggest that individuals with an internal LoC may enjoy higher levels of well-being because they invest more in social relationships compared to individuals with an external LoC.

**Table 4:** The effect of LoC on social capital.

	Social contacts	Volunteer/Charity work	Club member
LoC _t-1_	0.0162**	0.0129	0.00904***
	(0.0078)	(0.0089)	(0.0035)
Individuals	17,428	17,428	17,428
Observations	41,696	41,696	41,696

Note

**p<0.05

***p<0.01. Control variables include age, age squared, household size, real household income, educational attainment, employment status, marital status, negative life events, Australian states of residence and territories, and waves. Robust standard errors are in parentheses.

### 5.3 Testing for mediating effects

The findings reported so far indicate that individuals’ decisions to maintain an active lifestyle and to invest in social capital may partly explain the relationship between LoC and well-being. We can explore this possibility directly by conducting a mediation analysis in which the possible pathways are included as additional explanatory variables.

If the relationship between LoC and life satisfaction or mental health works indirectly through ‘third’ variables (so-called mediators), then we would expect that the estimated coefficient on LoC decreases once these variables are controlled for in the regression model. In addition, the proposed mediators would remain significant [32,33].

Column 1 of Table 5 shows estimated LoC coefficients when the possible mediators are not included in the model, while column 2 shows estimates on LoC when the mediators are accounted for. Looking across the columns, we can see that individuals’ perceptions of control continue to be significantly related to their life satisfaction and mental health. As expected, the estimated coefficients on LoC decrease in magnitude once the possible mediators are controlled for in column 2.

**Table 5:** Mediation analysis of LoC on well-being.

	Life satisfaction		Mental health	
	(1)	(2)	(1)	(2)
LoC _t-1_	0.111***	0.108***	0.118***	0.114***
	(0.0081)	(0.0080)	(0.0078)	(0.0077)
Physical activity		0.0630***		0.0916***
		(0.0068)		(0.0064)
Social contacts		0.0570***		0.0799***
		(0.0073)		(0.0065)
Club member		0.0308***		0.0199*
		(0.0118)		(0.0116)
Individuals	17,428	17,428	17,428	17,428
Observations	41,696	41,696	41,696	41,696

Note

*p<0.1

***p<0.01. Control variables include age, age squared, household size, real household income, educational attainment, employment status, marital status, negative life events, Australian states of residence and territories, and waves. Robust standard errors are in parentheses.

Table 6 summarizes the results of our mediation analysis. We note that our approach for quantifying the indirect effects of LoC on well-being is in line with the product-of-coefficients method (e.g., [32,34,50]). Consider, for example, the indirect effect of LoC on life satisfaction. This can be computed as the product of two coefficients: the estimate for LoC in the physical activity regression (Table 3, column 1) times the estimate for physical activity in the life satisfaction regression (Table 5, column 2); that is, 0.0322 × 0.063 = 0.002. To check whether the indirect effects are important in a statistical sense, a common approach is to use the Sobel test [50,51]. In our analysis we follow this approach to examine whether the indirect effects of LoC are indeed statistically significant.

**Table 6:** Estimates of the direct, indirect, and total effect of LoC on well-being.

	Life satisfaction	Mental health
(A) Indirect effect of physical activity	0.002***	0.0029***
	(0.0005)	(0.0007)
(B) Indirect effect of social contacts	0.0009**	0.0013**
	(0.0005)	(0.0006)
(C) Indirect effect of club member	0.0003*	0.0002
	(0.0002)	(0.0001)
(A + B + C) Total indirect effect	0.0032**	0.0044***
	(0.0013)	(0.0016)
(D) Direct effect	0.108***	0.114***
	(0.0080)	(0.0077)
(A + B + C + D) Total effect	0.1112***	0.1184***
	(0.0081)	(0.0079)
Total indirect/total effect	2.90%	3.74%
Individuals	17,428	17,428
Observations	41,696	41,696

Note

*p<0.1

**p<0.05

***p<0.01. (A), (B) and (C) are calculated by taking the effect of LoC on the mediators in Tables 3 and 4 and multiplying it with the effect of the corresponding mediators on well-being in Table 5. Significance of the indirect effects has been tested using the Sobel test (Sobel, 1982; Krull and MacKinnon, 2001). This test has been developed for mediation analysis and allows to examine whether the effect of LoC on well-being operates through the possible mediators.

Column 1 of Table 6 suggests that LoC has a significant direct and indirect impact on life satisfaction. The direct effect is positive, indicating that individuals with an internal LoC have higher levels of life satisfaction. The results also show that more frequent social interaction and more frequent participation in physical activity are two pathways through which LoC leads to higher well-being. The total indirect effect accounts for about 2.9% of the total effect. Physical activity is the largest contributor to the total indirect effect, explaining approximately 63% of its magnitude. Social contacts explain 28% of the total indirect effect.

Column 2 shows that, like life satisfaction, mental health is significantly explained by direct and indirect effects of LoC. The total indirect effect, the direct effect, and the total effect of LoC on mental health are all positive and statistically significant at p-values < 0.01. The direct effect increases mental health by 0.114 standard deviations. The share of the indirect effect in the total effect is approximately 3.74%. Physical activity continues to account for the main share of the total indirect effect at 66%, while social interaction accounts for 30%.

### 5.4 Gender subgroups

Previous research suggests that the effects of LoC are gendered (see e.g., [9,17,48]. To explore whether such differences matter within our mediation framework, we provide separate estimates for men and women. Looking across the columns of Table 7, we can see that the direct effect of LoC is still positive and statistically significant for both men and women. It is interesting to note that physical activity mediates the effect of LoC on life satisfaction and mental health among women and not men. Likewise, social interaction is a possible mediator only among women. To examine whether such differences do indeed matter in a statistical sense, we conducted a two-sample z-test. At the 95% confidence level, the null hypothesis of no gender differences can be rejected. Hence, in these data, men and women may differ in terms of the direct, indirect, and total effect of LoC on well-being.

**Table 7:** Estimates of the direct, indirect, and total effect by gender.

	Women		Men	
	Life satisfaction	Mental health	Life satisfaction	Mental health
(A) Indirect effect of physical activity	0.003***	0.0037***	0.001	0.0018*
	(0.0008)	(0.0010)	(0.0006)	(0.0011)
(B) Indirect effect of social contacts	0.0018***	0.0026***	-0.0001	-0.0001
	(0.0007)	(0.0009)	(0.0007)	(0.0009)
(C) Indirect effect of club member	0.0006*	0.0003	0.0000	0.0000
	(0.0003)	(0.0002)	(0.0001)	(0.0001)
(A + B + C) Total indirect effect	0.0053**	0.0065**	0.0009	0.0018
	(0.0024)	(0.0027)	(0.0009)	(0.0014)
(D) Direct effect	0.115***	0.118***	0.0943***	0.107***
	(0.0107)	(0.0105)	(0.0119)	(0.0113)
(A + B + C + D) Total effect	0.1203***	0.1245***	0.0952***	0.1088***
	(0.0110)	(0.0108)	(0.0119)	(0.0114)
Total indirect/total effect	4.44%	5.25%	0.98%	1.62%
Individuals	9,181	9,181	8,247	8,247
Observations	22,312	22,312	19,384	19,384

Note

*p<0.1

**p<0.05

***p<0.01.

### 5.5 Relaxing the cardinality assumption on well-being

The analysis to this point has used a linear specification. This assumes cardinality in the answers to the well-being questions, meaning that scores on the well-being scale are equidistant: the difference between a 3 and a 4 on the well-being scale, for instance, is the same as the difference between a 4 and a 5. Although the assumption of cardinality is consistent with the seminal work of [52], this approach has also been criticized by other scholars (see e.g., [53]).

To check the sensitivity of our results, we reconducted the mediation analysis by treating life satisfaction and mental health as ordinal variables. Accordingly, we estimated an ordered logit model with random effects: Table A2 in S1 Appendix reports the results. We can see that the direct effect of LoC continues to be positive and statistically significant at p-values < 0.01 for both men and women. Consistent with our main findings, we also observe significant mediating effects through physical activity and social interaction among women and not men, which lends further support for our empirical approach.

### 5.6 Using contemporaneous values of LoC

In our analysis we have used the value of LoC lagged to period *t*-1 in order to avoid the potential for reverse causality. While this assumption did not permit estimation of the contemporaneous effect of LoC, we noted that it may take some time for the effects of LoC to come into fruition and lead to higher well-being. Nonetheless, as the results from the transition matrix indicate, many people report a significantly different LoC score from one year to the next (see Table 1). Therefore, it could be argued that today’s LoC rather than last year’s LoC is most predictive of well-being today.

Table A3 in S1 Appendix probes this possibility. It does so by reconducting the mediation analysis using LoC at time *t*, rather than its lagged value, as the main explanatory variable of interest. Here, we find that physical activity and social interaction continue to have significant mediating effects among women. The importance of those mediators is further substantiated by the finding that they are now significant among men as well. In this regard, it is noteworthy that the total indirect effect, the direct effect, and the total effect of LoC have all become larger in magnitude. This lends further support for our conclusion that LoC matters for well-being both through indirect pathways and, more importantly, through a direct channel.

## 6. Concluding remarks

Using longitudinal data for Australia, we find that LoC has a significant direct and indirect impact on life satisfaction and mental health. The direct effect is positive, indicating that individuals with an internal LoC have higher levels of life satisfaction and mental health. We also show that physical activity and social interaction are two pathways linking an internal LoC to higher levels of well-being.

These findings can be useful for informing public policies. Given that a fixed effects model is used in our analysis, the estimated effects on well-being are all coming from within-person variation in LoC. In other words, it is a positive change in LoC during adulthood, rather than baseline LoC developed during childhood, that drives the implied positive effects on well-being. Thus, if the aim of policy is to improve well-being, the focus should be on enabling people to develop an internal LoC. So what we can practically do to help people increase their LoC?

In recent years, more and more attention has been given to developing interventions around strengths-based approaches (e.g., [54,55]). Such interventions are specifically designed to build personal strengths and resources necessary to create a meaningful life. In terms of fostering a more internal LoC, a strengths-based approach would encourage (i) learning to assume responsibility of the outcome of a situation rather than feeling that random events or someone else are to blame, and (ii) taking potential failure (that is often the byproduct of trying to accomplish something challenging) as an opportunity to learn from. Overall, such interventions may lead to higher well-being both through the identified channels and, more importantly, through the direct channel of LoC or via other unobserved pathways.

The study of the link between LoC and subjective well-being is likely to continue being a topic of intensive research in social sciences as many intriguing questions remain. For instance, does LoC affect satisfaction with different domains of one’s life, including job, income and personal relationships, and if so, what might help explain these relationships? These are just a few of the many more important questions that need answering, but our findings could shed new light on future directions that research on LoC and well-being might take.

## Supporting information

S1 Appendix (DOCX)
