# Supplementary material for: Locus of control and subjective well-being: Panel evidence from Australia
Source: PLoS One. 2022 Aug 31;17(8):e0272714. doi: 10.1371/journal.pone.0272714 (PMC9432765; doi:10.1371/journal.pone.0272714)
Supplement: S1 Appendix — (DOCX) [file pone.0272714.s001.docx]

**Appendix**


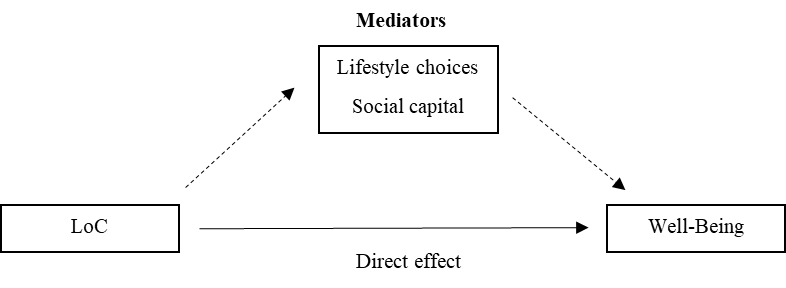


**Figure A1: The link between LoC and subjective well-being**

| **Table A1: Descriptive statistics** |  |  |  |  |
| --- | --- | --- | --- | --- |
| Variable | Mean | S.D. | Min | Max |
| Life satisfaction | 7.91 | 1.39 | 0 | 10 |
| Mental health | 74.47 | 16.94 | 0 | 100 |
| **Locus of control** |  |  |  |  |
| Little control | 2.67 | 1.57 | 1 | 7 |
| No ways to solve problems | 2.51 | 1.57 | 1 | 7 |
| Cannot change important things in life | 2.53 | 1.53 | 1 | 7 |
| Feel helpless | 2.47 | 1.55 | 1 | 7 |
| Pushed around | 2.56 | 1.59 | 1 | 7 |
| Future depends on me | 5.55 | 1.55 | 1 | 7 |
| Can do just about anything | 5.35 | 1.47 | 1 | 7 |
| **Control variables** |  |  |  |  |
| Age | 43.67 | 15.74 | 17 | 74 |
| Male | 0.46 | 0.50 | 0 | 1 |
| Household size | 2.89 | 1.42 | 1 | 17 |
| Real household income | 11.18 | 0.73 | 4 | 14 |
| College and above | 0.57 | 0.49 | 0 | 1 |
| **Marital status** |  |  |  |  |
| Legally married | 0.54 | 0.50 | 0 | 1 |
| Living as a couple | 0.14 | 0.34 | 0 | 1 |
| Separated | 0.03 | 0.16 | 0 | 1 |
| Divorced | 0.06 | 0.24 | 0 | 1 |
| Widowed | 0.02 | 0.15 | 0 | 1 |
| Never married and not living as a couple | 0.21 | 0.41 | 0 | 1 |
| **Employment status** |  |  |  |  |
| Employed | 0.69 | 0.46 | 0 | 1 |
| Unemployed | 0.03 | 0.17 | 0 | 1 |
| Not in the labor force | 0.28 | 0.45 | 0 | 1 |
| **Lifestyle choices** |  |  |  |  |
| Physical activity | 2.59 | 1.51 | 0 | 5 |
| Outdoor tasks | 3.95 | 5.89 | 0 | 80 |
| **Social interaction** |  |  |  |  |
| Social contacts | 3.46 | 1.46 | 0 | 6 |
| Club member | 0.38 | 0.48 | 0 | 1 |
| Volunteer/Charity work | 1.05 | 3.54 | 0 | 120 |
| **Negative life events** |  |  |  |  |
| Serious personal injury/illness | 0.08 | 0.27 | 0 | 1 |
| Serious injury/illness to family member | 0.16 | 0.36 | 0 | 1 |
| Death of spouse or child | 0.01 | 0.07 | 0 | 1 |
| Death of close relative/family member | 0.11 | 0.32 | 0 | 1 |
| Death of a close friend | 0.10 | 0.30 | 0 | 1 |
| Victim of physical violence | 0.01 | 0.11 | 0 | 1 |
| Victim of a property crime | 0.04 | 0.20 | 0 | 1 |
| Detained in jail | 0.00 | 0.04 | 0 | 1 |
| Close family member detained in jail | 0.01 | 0.12 | 0 | 1 |
| Fired or made redundant | 0.03 | 0.17 | 0 | 1 |
| Major worsening in finances | 0.03 | 0.17 | 0 | 1 |
| Note: The total number of observations is 41,696 across 17,428 individuals. | | | | |

| **Table A2: Estimates of the direct, indirect, and total effect using an ordered logit model with random effects** | | | | |
| --- | --- | --- | --- | --- |
|  | Women | | Men | |
|  | Life satisfaction | Mental health | Life satisfaction | Mental health |
| (A) Indirect effect of physical activity | 0.0217*** | 0.026*** | 0.0071 | 0.0136 |
|  | (0.0059) | (0.0068) | (0.0045) | (0.0083) |
| (B) Indirect effect of social contacts | 0.0142*** | 0.0204*** | 0.0001 | 0.0002 |
|  | (0.0049) | (0.0064) | (0.0046) | (0.0066) |
| (C) Indirect effect of club member | 0.0133* | 0.0045 | 0.0000 | 0.0000 |
|  | (0.0068) | (0.0052) | (0.0030) | (0.0031) |
| (A + B + C) Total indirect effect | 0.0492** | 0.0509* | 0.0082 | 0.0148 |
|  | (0.0246) | (0.0264) | (0.0080) | (0.0123) |
| (D) Direct effect | 0.325*** | 0.331*** | 0.265*** | 0.313*** |
|  | (0.0276) | (0.0269) | (0.0319) | (0.0307) |
| (A + B + C + D) Total effect | 0.3742*** | 0.3819*** | 0.2732*** | 0.3278*** |
|  | (0.0369) | (0.0377) | (0.0329) | (0.0331) |
| Total indirect/total effect | 13.15% | 13.32% | 2.99% | 4.50% |
| Individuals | 9,181 | 9,181 | 8,247 | 8,247 |
| Observations | 22,312 | 22,312 | 19,384 | 19,384 |
| Note: *p<0.1 **p<0.05 ***p<0.01. | | | | |

| **Table A3: Estimates of the direct, indirect, and total effect using contemporaneous values of LoC** | | | | |
| --- | --- | --- | --- | --- |
|  | Women | | Men | |
|  | Life satisfaction | Mental health | Life satisfaction | Mental health |
| (A) Indirect effect of physical activity | 0.0031*** | 0.0061*** | 0.0031*** | 0.0064*** |
|  | (0.0007) | (0.0009) | (0.0008) | (0.0010) |
| (B) Indirect effect of social contacts | 0.0038*** | 0.0062*** | 0.0026*** | 0.0036*** |
|  | (0.0008) | (0.0009) | (0.0008) | (0.0008) |
| (C) Indirect effect of club member | 0.0003 | 0.0002 | 0.0000 | 0.0000 |
|  | (0.0002) | (0.0002) | (0.0003) | (0.0001) |
| (A + B + C) Total indirect effect | 0.0072** | 0.0124*** | 0.0061** | 0.0101*** |
|  | (0.0031) | (0.0039) | (0.0030) | (0.0035) |
| (D) Direct effect | 0.239*** | 0.341*** | 0.203*** | 0.313*** |
|  | (0.0101) | (0.0093) | (0.0108) | (0.0098) |
| (A + B + C + D) Total effect | 0.2462*** | 0.3534*** | 0.2091*** | 0.3231*** |
|  | (0.0106) | (0.0101) | (0.0112) | (0.0104) |
| Total indirect/total effect | 2.92% | 3.52% | 2.90% | 3.14% |
| Individuals | 10,796 | 10,796 | 9,891 | 9,891 |
| Observations | 27,088 | 27,088 | 24,155 | 24,155 |
| Note: *p<0.1 **p<0.05 ***p<0.01. | | | | |
